# GCN-based stock relations analysis for stock market prediction

**DOI:** 10.7717/peerj-cs.1057

**Published:** 2022-08-11

**Authors:** Cheng Zhao, Xiaohui Liu, Jie Zhou, Yuefeng Cen, Xiaomin Yao

**Affiliations:** 1 School of Economics, Zhejiang University of Technology, Hangzhou, Zhejiang, China; 2 College of Computer Science and Technology, Zhejiang University of Technology, Hangzhou, Zhejiang, China; 3 School of Information and Electronic Engineering, Zhejiang University of Science & Technology, Hangzhou, Zhejiang, China; 4 College of Entrepreneurship, Zhejiang University of Technology, Hangzhou, Zhejiang, China

**Keywords:** Stock prediction, Multi-factor, Stock relation, Time series, Graph-based learning, LSTM

## Abstract

Most stock price predictive models merely rely on the target stock’s historical information to forecast future prices, where the linkage effects between stocks are neglected. However, a group of prior studies has shown that the leverage of correlations between stocks could significantly improve the predictions. This article proposes a unified time-series relational multi-factor model (TRMF), which composes a self-generating relations (SGR) algorithm that can extract relational features automatically. In addition, the TRMF model integrates stock relations with other multiple dimensional features for the price prediction compared to extant works. Experimental validations are performed on the NYSE and NASDAQ data, where the model is compared with the popular methods such as attention Long Short-Term Memory network (Attn-LSTM), Support Vector Regression (SVR), and multi-factor framework (MF). Results show that compared with these extant methods, our model has a higher expected cumulative return rate and a lower risk of return volatility.

## Introduction

According to the statistics of the World Bank, in 2021, the ratio of global stock market value to GDP has exceeded 130% (https://data.worldbank.org.cn/indicator/CM.MKT.LCAP.GD.ZS), which means that the stock market has become one of the most popular investment channels. As defined in the capital asset pricing model (Fama & French, 2004), the returns obtained by an investor in the stock market mainly comprise two parts: alpha returns (the part of individual returns that does not fluctuate with the market) and beta returns (the part of systematic returns that follows market fluctuations). The former depends on a company’s long-term operation, the analysis of which is best performed by humans. In contrast, the latter fluctuates sharply with the market in the short term, which is more suitable for technical analysis using quantitative models. Based on stock price characteristics, quantitative models search for undervalued stocks and profit by their value return.

Traditionally, quantitative models based on deep learning treat the transaction data of a single stock as a time series (Cao, Li & Li, 2019; Bao, Yue & Rao, 2017; Narayan, Phan & Liu, 2021; Greenwood & Shleifer, 2014; Zhang, Ashuri & Deng, 2017; Dai, Shao & Lu, 2013; Kazem et al., 2013). The recurrent neural network (RNN) and its various variants that are good at modeling sequence achieved good performance in stock price prediction (Bao, Yue & Rao, 2017; Cao, Li & Li, 2019; Chen & Ge, 2019; Fischer & Krauss, 2018; Pang et al., 2020; Zhang, Aggarwal & Qi, 2017; Liu, Li & Liu, 2021). However, they treat stocks as separate entities, ignoring their relations and the integrity of the stock market.

**Figure 1:**
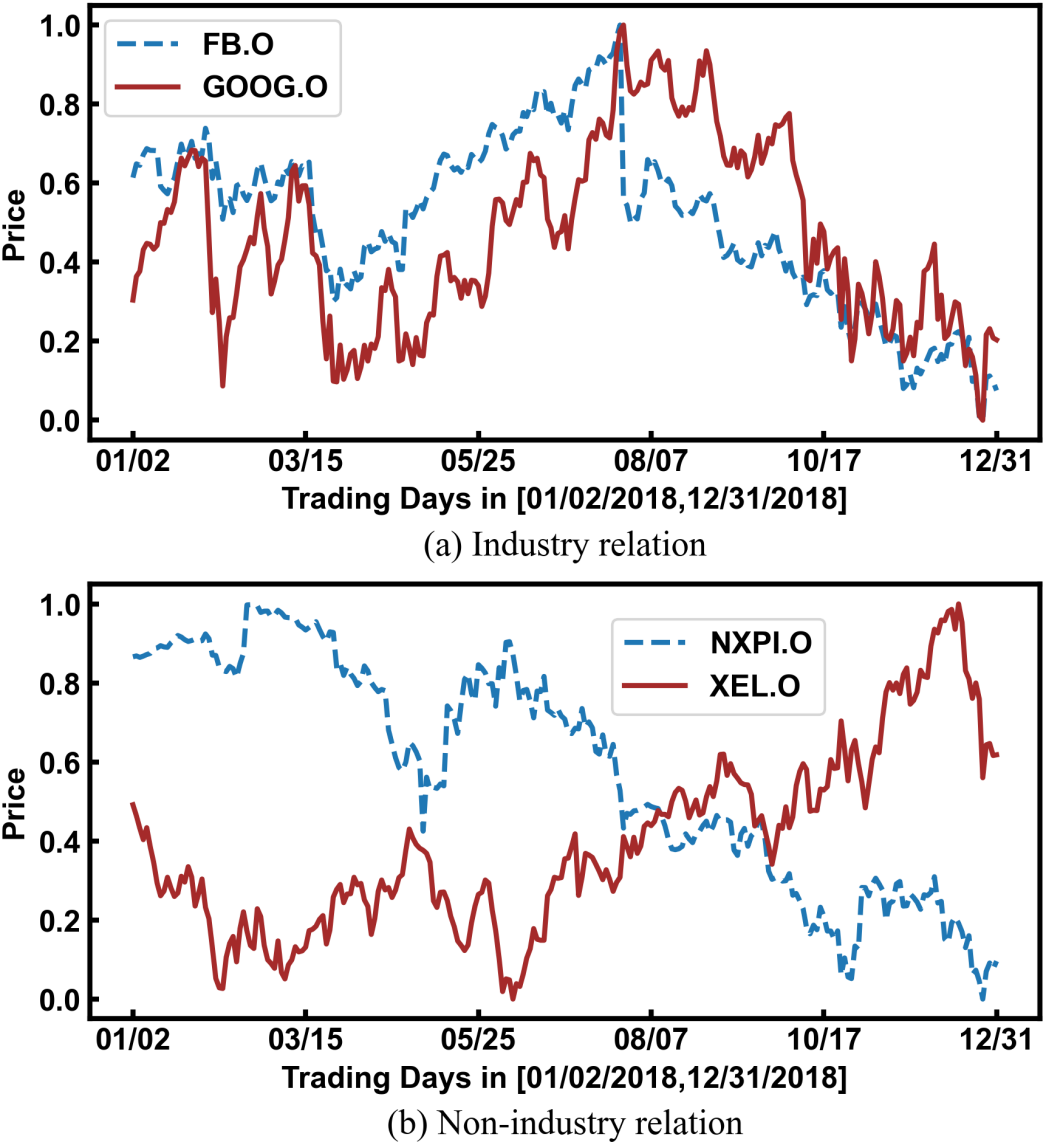
The impact of stock relations on stock prices. The horizontal axis represents a period of trading time, and the vertical axis represents the normalized stock price.

In fact, the prices of related stocks significantly impact each other. As shown in Fig. 1A, Facebook and Google belong to the same industry, and their stock prices show the same trend. Therefore, stock relations have been used to enhance the prediction accuracy with the development of graph learning. However, most researchers directly and simply use industry relations provided by third-party platforms, which are insufficient. As shown in Fig. 1B, the stock prices of NXPI and XEL have shown opposite trends for a long time. Because they represent the new energy vehicle industry and the traditional fossil energy industry, respectively, there is a competitive relationship between them. However, third-party platforms cannot provide such complex relationships.

The motivation of the study is to solve the problems of incomplete information and insufficient stock relations in the existing stock price prediction methods mentioned above. Therefore, in the paper, we design the algorithm to mine stock relations more effectively and integrate them with multiple information to improve the prediction. The key contributions include: (1) We designed the self-generating relations algorithm (SGR) that uses trading data to generate stock relations. Meanwhile, we modified the traditional graph convolutional neural network (GCN) to model the influence of stock relations on price; (2) We proposed the time series relational multi-factor model (TRMF) model that combines multiple dimension information for stock price prediction; (3) We demonstrated the effectiveness and universality of TRMF with data from the two most developed stock markets, New York Stock Exchange (NYSE) and NASDAQ Stock Market (NASDAQ).

The remainder of this article is organized as follows: the “Related Work” section reviews research on the technical analysis of stock price predictions and stock relations; then the “Method” section details the structure of the TRMF model, especially the SGR algorithm and the weighted graph convolutional neural network (WGCN) module; in the “Experimental Setting” section, experimental data and model parameters are listed; furthermore, the “Results” section presents the back-testing results of the proposed model and its comparison with other models; finally, the “Conclusion” section concludes the effectiveness of the SGR algorithm and the TRMF model on the stock price prediction.

## Related Work

There is a linkage effect between the stocks in the market, which has been widely proved in academics (Baruník & Křehlík, 2018; Diebold & Yilmaz, 2014; Ferrer et al., 2018; Ma et al., 2022; Nasreen et al., 2020; So, Chu & Chan, 2021). Among them, Diebold & Yilmaz (2014) proposed several connectedness measures built from pieces of variance decompositions and tracked daily time-varying connectedness of major US financial institutions’ stock return volatilities on the financial crisis of 2007–2008. Ferrer et al. (2018) demonstrated the time and frequency dynamic connectedness among oil prices, stock returns of American clean energy companies and several vital financial variables with the method proposed by Baruník & Křehlík (2018). Nasreen et al. (2020) analysed the dynamics of connectedness between fossil energy prices and stock market returns of clean energy and technology companies. The results demonstrated that the connectedness volatility was transmitted at all frequencies and over the whole experimental period. So, Chu & Chan (2021) studied the impacts of the COVID-19 pandemic on the connectedness of the Hong Kong financial market. Compared to other crises where the network density and clustering can be explained by co-movement with market indices as in normal periods, they found that both network density and clustering were higher in the partial correlation networks during the COVID-19 outbreak. Despite the extensive literature demonstrating the universality of connectedness among stocks, quantitative models do not take them into account.

Traditionally, researchers treat stock price prediction as a time-series problem and solve it with classic RNN models such as LSTM. Existing researches show that LSTM can effectively extract time-series information and has good performance in stock price prediction (Cao, Li & Li, 2019; Chen & Ge, 2019; Fischer & Krauss, 2018; Kim & Won, 2018; Zhang, Aggarwal & Qi, 2017; Bao, Yue & Rao, 2017; Tang et al., 2020; Rezaei, Faaljou & Mansourfar, 2021; Zhao et al., 2021). For example, Fischer & Krauss (2018) made an earlier attempt to use LSTMs for stock selection of S&P 500 constituents based on the time series characteristics. The experimental results found LSTM networks to outperform memory-free classification methods, *i.e.,* a random forest (RAF), a deep neural net (DNN), and a logistic regression classifier (LOG). Cao, Li & Li (2019) proposed two hybrid forecasting models which combine the two kinds of empirical mode decomposition (EMD) with LSTM to improve the accuracy of the stock market prices forecasting. Compared with the single LSTM model, support vector machine (SVM), multi-layer perceptron (MLP), and other hybrid models, the experimental results showed that the proposed models displayed better performance in one-step-ahead forecasting of financial time series. Similarly, Zhang, Aggarwal & Qi (2017) proposed the State Frequency Memory (SFM) recurrent network, which decomposed the hidden states of memory cells into multiple frequency components. Each component models a specific frequency of latent trading patterns. Kim & Won (2018) combined the LSTM model with various generalized autoregressive conditional heteroscedasticity(GARCH)-type models for stock price prediction. They discovered that the hybrid model combining the LSTM model with three GARCH-type models had the best performance in terms of many metrics. Currently, researchers have found that the attention mechanism exhibits excellent performance in long sequence learning (Vaswani et al., 2017; Yu et al., 2021). Therefore, Chen & Ge (2019) explored the attention mechanism in LSTM network-based stock price movement prediction. The experimental results in Hong Kong stock movement prediction demonstrated the effectiveness. However, as mentioned above, most of these traditional solutions do not incorporate stock relations into the predictive models.

Incorporating stock relations into stock price prediction is a relatively new research direction (Feng et al., 2019; Chen, Wei & Huang, 2018; Long et al., 2020). Typically, Feng et al. (2019) obtained the industry classification data of the NASDAQ and NYSE markets in the United States from the website (https://www.nasdaq.com/screening/industries.aspx) to construct a graph and input it to the GCN. The experimental results showed that this method outperformed the traditional LSTM model in backtesting returns. In addition, Chen, Wei & Huang (2018) obtained the stock relations from the Wind. (https://www.mediawiki.org/wiki/Wikibase/DataModel/JSON), and used the relationship transfer method to increase the number of stock relations. The experimental results demonstrated that increasing the number of stock relations can improve the performance of the prediction. Nonetheless, third-party platforms can only provide industry relations, which are not comprehensive enough.

Scarselli et al. (2009) firstly proposed GCN, extending deep neural networks to graph category. Years later, Bruna et al. (2014) performed convolution operations on the frequency domain to capture the local connection patterns in graphs. Based on their method, several works have been done for accelerating (Defferrard, Bresson & Vandergheynst, 2016; Kipf & Welling, 2017). Among them, Kipf & Welling (2017) simplified GCN *via* local first-order approximation based on spectral graph convolutions and demonstrated that the method outperformed existing methods in experiments on citation network and knowledge graph datasets. GCN is used for single relational networks between entities (Wu et al., 2020; Fout et al., 2017; Zhao et al., 2020). However, there may be multiple relations between two stocks. For example, a machine tool manufacturer A may use the bolts provided by enterprise B. At the same time, A supplies B with machine tools for producing bolts. Then there are two relations between A and B: A is both the downstream shipper of B and the supplier of B. Therefore, the classical GCN needs to be adapted to the characteristics of enterprise relations for stock price prediction.

## Method

Acquiring and utilizing stock relations is the critical component of the TRMF model. In this article, our proposed SGR algorithm uses transaction data to obtain stock relations. Meanwhile, the WGCN module models the impact of relationships on the stock price prediction.

### Self-generating relation algorithm

We consider determining whether relations exist between two stocks and how to design rules to capture them. Here, we refer to realistic stock relations as entity relations, such as industrial relations and competitive relations. A stock may have entity relations with many other stocks. The combined relations ultimately act on stock pairs’ trading behavior, especially prices in different periods. So that their prices show a specific pattern, such as two stocks’ closing prices rising or falling together or one stock’s five-day average price rising while another falls. Therefore, the correlation between two stock prices reflects a combination of multiple relations between them. There is a mapping:

(1) \begin{eqnarray*}({R}_{i,j}^{1},{R}_{i,j}^{2},\ldots ,{R}_{i,j}^{N})\rightarrow _{}^{f}{r}_{i,j}^{k}\end{eqnarray*}

where *R*_*i*,*j*_ denotes an entity relation between stock *i* and stock *j*, ${r}_{i,j}^{k}$ denotes the correlation between stock *i* and stock *j* in certain trading data. Both are Bool data, represented by 0 and 1.


 
 
 Data: stocks S, trading days Tr, trading features F, minimum support 
              min_support 
    Result:  Stock relation adjacency matrix Asgr[len(S)][len(S)][len(F)] 
  1  Function gen_Stocks_Feature_Event(feature): 
     2   Event_Bit_Set[len(S)][len(Tr)] = {0} 
3   for t in Tr do 
    4   for s in S do 
5   if trading feature of stock s in day t meet feature threshold 
   then 
6   Event_Bit_Set[s][t] = 1 
7   end 
8   end 
9   end 
10   return Event_Bit_Set 
11 
12  for f in F do 
     13   E = gen_Stocks_Feature_Event(S, Tr, f) 
14   for (s1,s2) pair from S do 
    15   dis = Hamming Distance between E[s1] and E[s2] 
16   support = (len(Tr) - dis) / len(Tr) 
17   if support >min_support then 
18   Asgr[s1][s2][f] = 1 
19   else 
20   Asgr[s1][s2][f] = 0 
21   end 
22   end 
23  end 
 
              Algorithm 1: Self-generating relation algorithm    


Therefore, we use the correlation of stock prices, namely self-generating relations (SG-relations), as an alternative to entity relations. The SGR algorithm mines stocks’ SG-relations. Its input is multiple trading features **F**, and the output is a 3D adjacency matrix **A**_sgr_. The flow of the algorithm is represented in pseudocode in Algorithm 1. In short, two stocks have a SG-relation on a trading feature, which means they meet the same threshold on the feature for enough days. The threshold here can be similar to that the closing price is greater than 0, the five-day average price is less than 0, and so on.

### Weighted graph convolutional neural network

GCN requires two inputs: an adjacency matrix **A** representing the relationship, and the other is a matrix **H** representing stocks’ time-series features. We use a simplified GCN proposed by Kipf & Welling (2017) which is the state-of-the-art formulation (Chen, Wei & Huang, 2018; Feng et al., 2019). It consists of two convolutional layers, one for input-to-hidden and the other for hidden-to-output:

(2) \begin{eqnarray*}\mathbi{A}=\mathbi{D}-1/2(\mathbi{A}+\mathbi{I})\mathbi{D}-1/2\mathbi{Y }=f(\mathbi{X},\mathbi{A})=softmax \left( \mathbi{A} \left( ReLU(\mathbi{A}\mathbi{X}\mathbi{W}0) \right) \mathbi{W}1 \right) \end{eqnarray*}

Here, **A** + **I** is the adjacency matrix of stock relations undirected graph **G** with added self-connections, **D** ∈ ℝ^*N*×*N*^ is the degree matrix of **G**.**W**0 ∈ ℝ^*C*_1_×*C*_3_^ is an input-to-hidden weight matrix, and **W**1 ∈ ℝ^*C*_3_×*C*_4_^ is the hidden-to-output weight matrix.

Note that **A** is a 2D adjacency matrix, which means only a single relation between stock nodes. However, two stocks may have multiple SG-relations as defined in the previous section. Therefore, we designed the WGCN to utilize relational information. It adds a weight layer to the traditional GCN to map various SG-relations into a synthetic relation:

(3) \begin{eqnarray*}\mathbi{A}{sgr}^{(N,N,{n}_{r})}\rightarrow \mathbi{W}{r}^{({n}_{r},1)}\mathbi{A}(N,N)\mathbi{A}=\mathbi{W}r\mathbi{A}sgr\end{eqnarray*}

where **A**_*sgr*_ denotes the adjacency matrix of multiple SG-relations, and **W**_*r*_ denotes the weight vector.

**Table B1:** Evaluation metrics. Here, *o*_*t*_ is open price of day *t*, *p* is the principal. *r*_*up*_ is a local high point of return ratio and *r*_*down*_ is a local low point. *r*_*p*_ is return of portfolio, *r*_*f*_ is risk-free rate, and *δ*_*p*_ is standard deviation of the portfolio’s excess return.

Metrics	Formula
IRR	${\mathop{\sum }\nolimits }_{t=1}^{n} \frac{{o}_{t}-{o}_{t-1}}{p} $
MDD	$\max ( \frac{{r}_{up}-{r}_{down}}{{r}_{up}} )$
SR	$ \frac{{r}_{p}-{r}_{f}}{{\delta }_{p}} $

**Figure 2:**
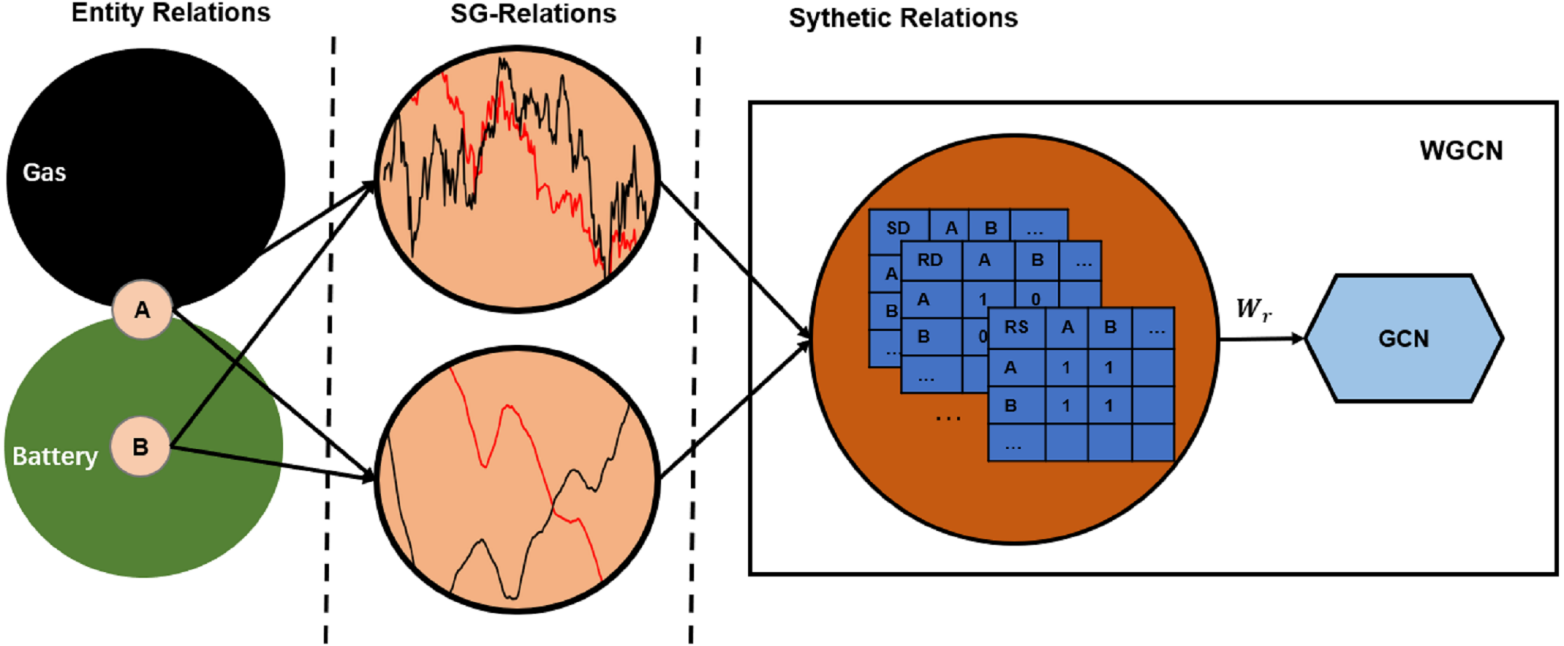
Synthetic relations generation. SD, SS, and RS are short for “daily price in the same trend”, “60-day average price in the same trend”, and “60-day average price in the reverse trend”, respectively. The complex relationship between companies A and B causes their closing prices to have the same trend, while the 60-day average price has the reverse trend. Therefore, their intersection on adjacency matrices SD and RS is 1, and on RD is 0.

Figure 2 illustrates mapping the entity relations to a synthetic relation. Suppose that A is a traditional auto company actively turning to new energy, and B is a power battery company. They used to have mutually exclusive competition and are gradually increasing cooperation. These complex entity relationships are reflected in market transactions, which may be the reverse trend of the 60-day average price because it reflects longer periods. It may also show the same trend of the daily trading price because it reflects the latest situation. These SG-relations are combined by weights to form a synthetic relation that is fed into the GCN.

### Framework in whole

**Figure 3:**
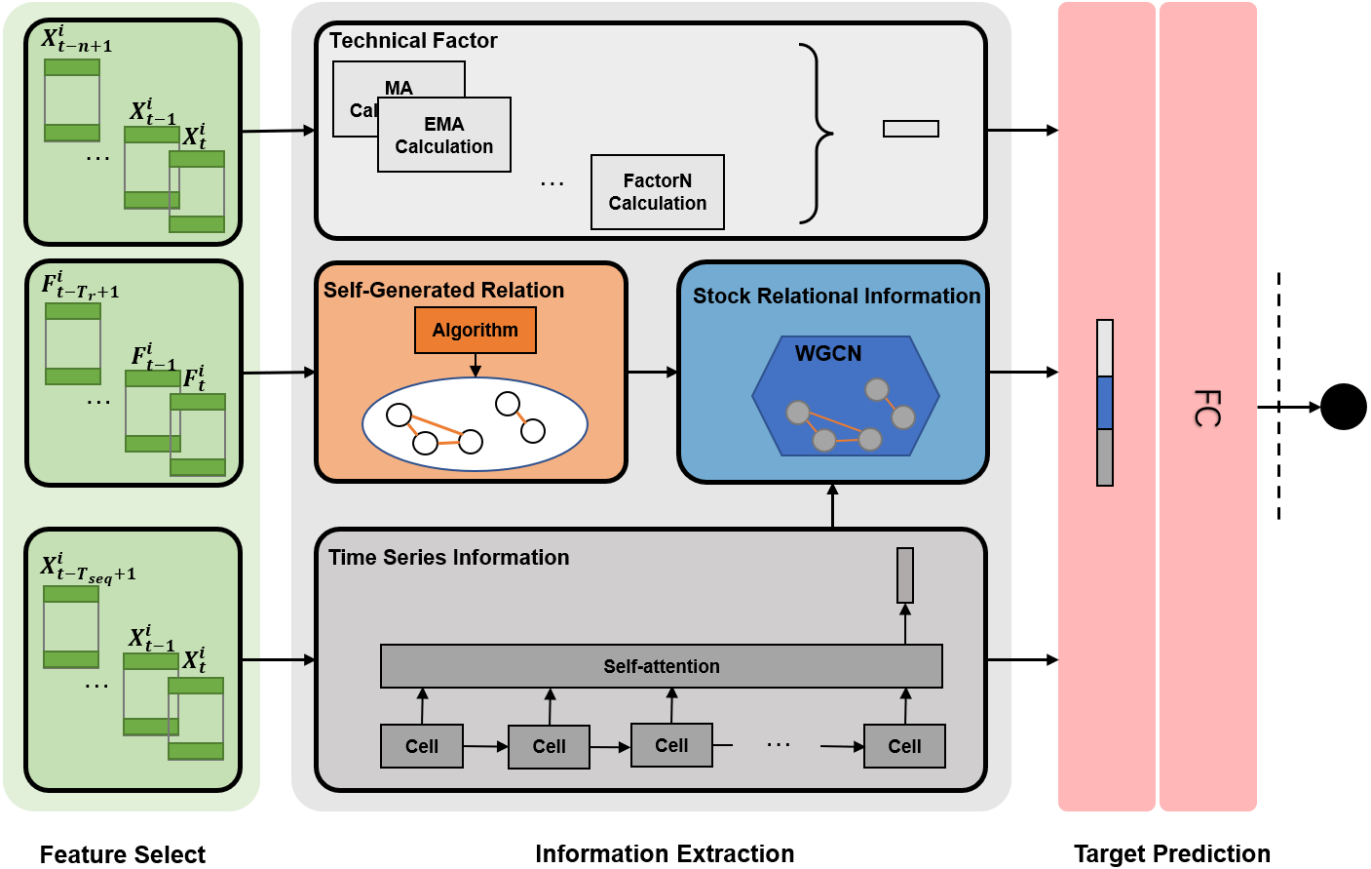
TRMF model. Here, **T**_**r**_ is the period for generating relations, **T**_**seq**_ is the period of the time series, *n* is the parameter of specific technical factors, ${\mathbf{X}}_{\mathbf{t}}^{\mathbf{i}}$ denotes stock features of stock *i* in *t* day, and ${\mathbf{F}}_{\mathbf{t}}^{\mathbf{i}}$ denotes trading features used for mining relationships. **FC** means a fully connected layer.

The TRMF model comprises feature selection, information extraction, and target prediction. Figure 3 shows the framework in whole.

#### Feature selection

We first determine three elements: stocks, experimental time, and trading features to get the original data and then filter it. Drawing on the practice of Feng et al. (2019), we perform two conditions to filter the stocks in the market: during the experimental period, (1) the actual trading day percentage greater than 98%; and (2) stock price not less than 15 dollars. The first condition concerns that too many suspension days could damage the statistical characteristics of the data and that the model can learn abnormal patterns. The second condition is to avoid penny stocks (https://www.sec.gov/fast-answers/answerspennyhtm.html) which are risky for general investors, as suggested by the U.S. Securities and Exchange Commission. For the suspension day, the stock trading data maintained the previous day’s record.

#### Information extraction

We divide the information reflecting stocks into three categories: technical factors, time-series information, and relationship impact factors. Among them, the technical factor shows stocks’ financial statistics, the time series information captures the historical sequence pattern, and the relational factor analyzes the impact of the market environment on the stock. The TRMF model combines them to predict stock prices.

*Technical Factor*: We pick up five mature and common technical factors: Moving Average (MA), Exponential Moving Average (EMA), Relative Strength Index (RSI), Chande Momentum Oscillator (CMO), On balance volume(OBV) (Fama & French, 2015; Nazemi, Heidenreich & Fabozzi, 2018; Wang, Zhuang & Feng, 2022).

*Time Series Information*: We use an Attn-LSTM to extract the stocks’ time-series information. LSTM is an excellent variant of the RNN, which uses a four-layer neural network in a recurrent unit to solve the problem of gradient disappearance and explosion during long-sequence training. Therefore, LSTM can outperform an ordinary RNN in longer sequences. It can be represented in short as:

(4) \begin{eqnarray*}\mathbi{h}ti=LSTM(\mathbi{x}ti,\mathbi{h}t-1i),\end{eqnarray*}

where ${\mathbf{x}}_{\mathbf{i}}^{\mathbf{t}}$ and ${\mathbf{h}}_{\mathbf{i}}^{\mathbf{t}}$ denote stock features and hidden layer states of stock *i* on *t* day.

In addition, specific time points often relate to stock price movements, such as when enterprises release their quarterly financial reports or launch new products. Therefore, we use the enhanced LSTM model with attention. The attention mechanism assigns different weights to time series so that the model can pay attention to a particular critical trading day.

(5) \begin{eqnarray*}\mathbi{u}ti=\tanh \nolimits (\mathbi{W}a\mathbi{h}ti)\end{eqnarray*}

(6) \begin{eqnarray*}\boldsymbol{\alpha }t,{t}^{{^{\prime}}}i= \frac{\exp \nolimits (\mathbi{u}it\top \mathbi{u}i{t}^{{^{\prime}}})}{\sum _{j=1}^{T}\exp \nolimits (\mathbi{u}it\top \mathbi{u}ij)} \end{eqnarray*}

(7) \begin{eqnarray*}\tilde {\mathbi{h}t}i=\sum _{t{^{\prime}}=1}^{T}\boldsymbol{\alpha }t,t{^{\prime}}i\top \mathbi{u}t{^{\prime}}i\end{eqnarray*}

where *T* is the length of time series and **W**_a_ ∈ℝ^*C*_2_×*C*_1_^ is a randomly initialized matrix, optimized during training. $\tilde {\mathbf{h}}$${}_{i}^{t}$ is the hidden layer state of Attn-LSTM, which describes the time-series features of stock *i*.

*Relationship Impact Factor*: The critical component of using the relationship to predict stock prices is acquiring and utilizing it. As described above, we used the SGR algorithm to mine stock relations and WGCN to obtain relationship impact factors.

#### Target prediction

Finally, all three kinds of information were spliced together and inputted into the fully connected layer.

(8) \begin{eqnarray*}{z}^{i}=ReLU(\mathbi{w}[\mathbi{fi}:{\tilde {\mathbi{h}}}^{i}:\mathbi{yi}]^{\top }+b)\end{eqnarray*}

where **f***i* is a vector composed of technical factors, **y***i* is the relational impact factor from the WGCN model. *z*^*i*^ is the expected return of stock *i* on the next day, expressed here as *o*_*t*_/*o*_*t*+1_.*o*_*t*_ is the opening price of *t* day.

## Experimental Setting

### Experimental data

Experiments data is from the New York Stock Exchange (NYSE) and the Nasdaq Stock Exchange (NASDAQ). Because they are the top two stock markets in terms of global market capitalization in 2021 (https://data.worldbank.org/indicator/CM.MKT.LCAP.CD), and the most developed markets in the financial industry in the world. Then the constituent stocks of the S&P 500 are chosen for stock selection. This method, on the one hand, reduces the amount of calculation. On the other hand, these stocks contain 80% of the US stock market value (https://en.wikipedia.org/wiki/S%26P_500), so they can represent the entire market.

The experimental period was 01/02/2018–06/28/2019 and was divided into three intervals: the training (01/02/2018–12/28/2018), validation (01/02/2019–03/29/2019), and test (04/01/2019–06/28/2019) sets. We made this choice because S&P 500 Index shows periods of volatility, plummeting, and soaring in the test set, which is representative. Table 1 lists the number of trading days in the NYSE and NASDAQ.

**Table 1:** Experimental date.

Market	Time range	Train	Validation	Test
	01/02/2018	01/02/2018	01/02/2019	04/01/2019
	06/28/2019	12/28/2018	03/29/2019	06/28/2019
NYSE	349	235	56	58
NASDAQ	349	235	56	58

We screened 134 stocks on the NASDAQ and 325 on the NYSE during the experimental period based on the two filters mentioned above. Table 2 shows the number of stocks used in the experiment and Appendix lists all stocks.

**Table 2:** Statistics of filtered stocks.

Index	Market	All	Condition 1	Condition 2	Final
S&P 500	NYSE	355	13	17	325
	NASDAQ	150	7	9	134

We collected the opening price, closing price, highest price, lowest price, trading volume, and turnover rate as stock features. Because they are typical and common, and any stock exchange will provide them. Furthermore, all data were normalized to [0,1] using Min-Max normalization.

We selected four trading features: closing price, 5-day moving average, 20-day moving average, and 60-day moving average as input of SGR algorithm to generate SG-relations. They represent the stock’s trading behavior on the day, week, month, and quarter, respectively. Each trading feature has two different forms of correlation: the same trend and the reverse trend. So, there is eight SG-relations in total. Table 3 list all SG-relations.

**Table 3:** SG-relations.

SG-Relation	Event	Minimum support
SD	the closing price of stock A and stock B changed in the same trend	0.6
RD	the closing price of stock A and stock B changed in the reverse trend	0.6
SW	the 5-day average price of stock A and stock B changed in the same trend	0.6
RW	the 5-day average price of stock A and stock B changed in the reverse trend	0.6
SM	the 20-day average price of stock A and stock B changed in the same trend	0.6
RM	the 20-day average price of stock A and stock B changed in the reverse trend	0.6
SS	the 60-day average price of stock A and stock B changed in the same trend	0.6
RS	the 60-day average price of stock A and stock B changed in the reverse trend	0.6

### Trading strategy

We used a daily cycle “buy-hold-sell” trading strategy to simulate market investments, which has been commonly used in many articles (Feng et al., 2019; Chen & Ge, 2019; Fischer & Krauss, 2018). On each *t* + 1 day of the test time set, at the opening moment, we simulated a trader selling out all stocks held at the opening price (buy on *t* day) and then buying the stock with the highest expected revenue.

Transaction rates are ignored (Feng et al., 2019).

### Evaluation metrics

Our purpose was to predict stock prices accurately and balance the return and risk of stock investment. So we employed the following four metrics to evaluate and compare the models: mean square error (MSE), cumulative investment return ratio (IRR), maximum drawdown (MDD), and Sharpe ratio(SR) (Wang, Zhuang & Feng, 2022; Feng et al., 2019; Deng et al., 2016). The detailed formulas are presented in the Table B1 in the Appendix B.

MSE has been a standard evaluation metric for regression tasks in machine learning. Since directly reflecting the effect of stock investment, IRR is our primary metric. It is calculated by summing the return ratios of the selected stock on each testing day. MDD describes the worst possible situation in the investment process, which significantly affects investors’ pessimism. It is a significant risk metric. Finally, SR comprehensively evaluates the performance of an investment behavior from the two dimensions of return and risk and is familiar to investors. It should be noted that the *r*_*f*_ and *δ*_*p*_ in the SR formula are very sensitive to the calculation frequency. This study performs the calculation daily since back-testing uses the daily “buy-hold-sell” strategy. Smaller MSE and MDD values and larger IRR and SR values indicate better performance.

### Experimental models & parameter settings

Table 4 shows all compared models in back-testing experiments. It is worth emphasizing that TGC is the main one because it incorporates industry relations from third-party platform into stock predictions and is the state-of-the-art stock relation-based solution. Moreover, its source code and data are open access (Feng et al., 2019).

**Table 4:** Experimental models.

Model	Description
Five-Factors	Traditional multi-factor model
SVR	Multi-factor model based on machine learning
Attn-LSTM	Make use of time series information
TGC	Integrate time series information and industry relations
WGCN	Make use of SG-relations
TRMF	Integrate technical factors, time series information and SG-relations
Baseline	DJIA in NYSE and IXIC in NASDAQ

We draw on previous literature to select the hyperparameters for existing models (Dai, Shao & Lu, 2013; Chen & Ge, 2019; Feng et al., 2019; Liu, Liu & Zhang, 2022). In particular, for SVR, Attn-LSTM and TGC, we follow the original setting in Dai, Shao & Lu (2013); Chen & Ge (2019) and Feng et al. (2019), respectively. For all deep learning methods, we apply the Adam optimizer with a learning rate of 0.001. Furthermore, We employ grid search to select the optimal hyperparameters regarding IRR for other models. We tune two hyperparameters for Attn-LSTM layer in TGC, the length of sequential input *T*_*seq*_ and hidden units *C*_1_ within {5, 10, 20, 60} and {32, 64, 128}, respectively. Besides *T*_*seq*_ and *C*_1_, we further tune the length of trading days *T*_*r*_ in SGR layer. Specifically, we tune *T*_*r*_ within {20, 60, 120, 240}. We futher tune *C*_3_ and *C*_4_ in WGCN layer within {64, 128, 256} and {64, 128, 256}, respectively.

Finally, the specified parameters of our trained TRMF model are as follows:

• Input layer with *T*_*seq*_ = 20, which corresponds to a trading month.
• LSTM layer with *C*_1_ = 64 hidden neurons.
• SGR layer with *T*_*r*_ = 240, which corresponds to a trading year.
• WGCN layer with *C*_3_ = 128 input-to-hidden neurons and *C*_4_ = 128 hidden-to-output neurons.
• Output layer with one neuron and *ReLU* activation function.

## Results

Our study aims to generate and use more effective stock relations and integrate them for stock market forecasting. Therefore, we conducted experiments to answer two research questions: First, can our proposed SGR algorithm effectively extract relation information among stocks? Second, can the proposed TRMF model effectively integrate multi-dimension information and predict the stock market better than previous researches in both return and risk?

### Study of SG-relations

We use the proposed SGR algorithm to mine SG-relations in the NASDAQ and NYSE markets. Then, an experiment will compare SG-relations with the industry relations. Industry relations are extracted from sector-industry data maintained by NASDAQ Inc (https://www.nasdaq.com/screening/industries.aspx). Feng et al. (2019) used them in their TGC model and presented in its appendix.

**Figure 4:**
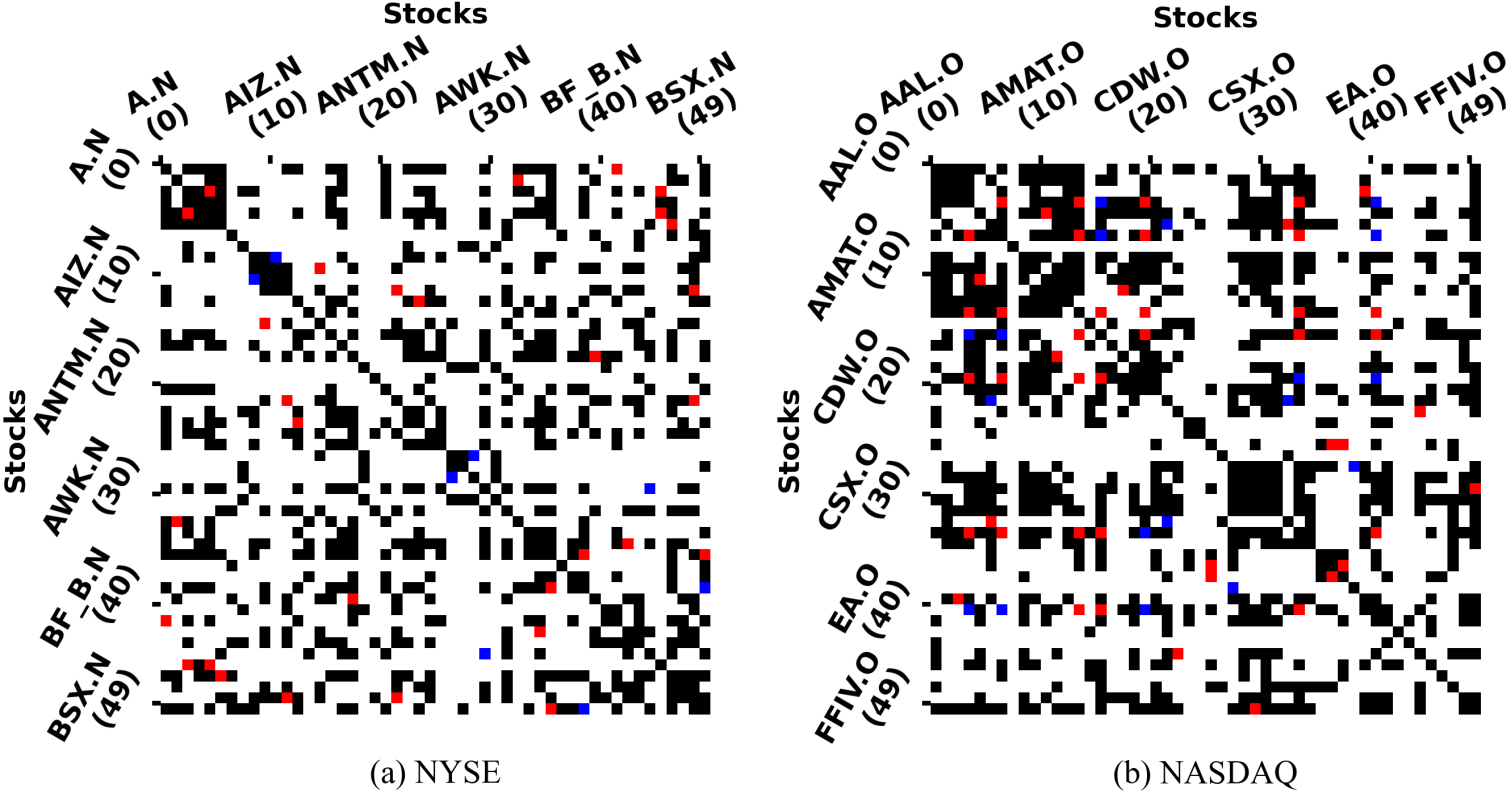
SG-relations and industry relations in NYSE & NASDAQ. The horizontal axis is the index of 50 stocks, and so is the vertical axis. The color of the intersection indicates whether there is a relationship between the two stocks. The white point indicates that two stocks have neither a SG-relation nor a industry relation. In contrast, the red point indicates that both relations exist, and the black point with only SG-relation while the blue point with only industry relation. The figure just contains top-50 stocks because the number of stocks in each market is different and too large.

Figure 4 qualitatively presents the coverage of SG-relations and industry relations *via* the distribution of intersection colors. Firstly, we can see that the number of red points representing coverage far exceeds the number of blue points representing non-coverage, so SG-relations can largely cover industry relations. Furthermore, a large number of black points are scattered across the graph, which indicates that the SGR algorithm is able to expand the industry relations.

**Figure 5:**
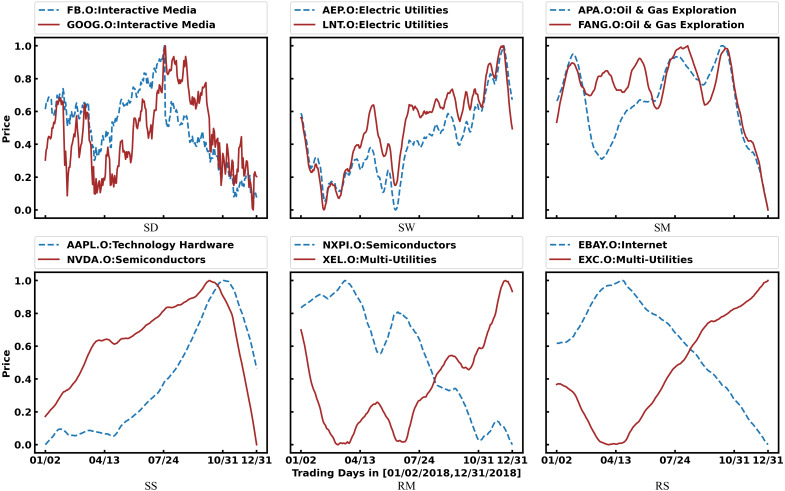
Examples of SG-Relations. As defined in Table 3, SD, SW, SM, and SS mean daily, 5-day, 20-day, and 60-day average prices in the same trend, respectively. Also, RM and RS mean 20-day and 60-day average price in the reverse trend. Furthermore, the industry to which the stock belongs is marked after the legend.

Figure 5 shows examples of SG-relations. Among them, we can see that SG-relations can cover many industry relations (https://en.wikipedia.org/wiki/List_of_S%26P_500_companies), such as FB.O & GOOG.O, AEP.O & LNT.O, and APA.O & FANG.O. Furthermore, there are several non-industry relations in SG-relations. For example, NVDA.O is the supplier of AAPL.O. Their 60-day average prices show the same trend, yet AAPL.O slightly lags behind NADA.O. In addition, NXPI.O has an exclusive relation with XEL.O because the former represents the new energy vehicle industry while the latter represents the traditional fossil energy industry. Their 20-day average prices fluctuate reversely. Finally, EBAY.O’s 60-day average price shows an apparent reverse trend with EXC.O’s for a long time while we cannot figure it out by industry analysis. There may be some internal connection between them. This information can help predict their price more accurately.

**Figure 6:**
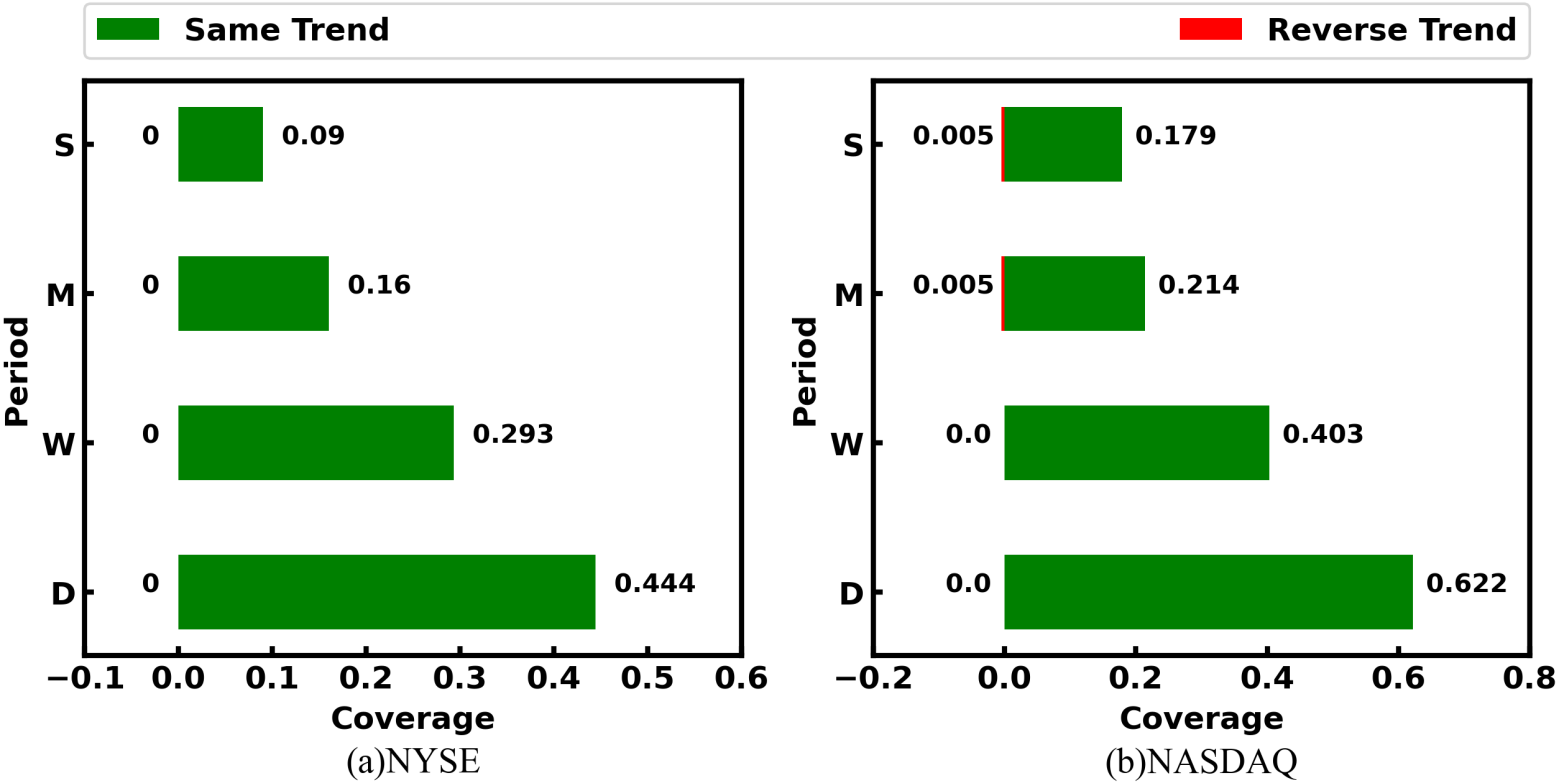
Coverage ratio of SG-relations to industry relations. The labels in the vertical ordinate - D, W, M, and S denote day, week, month, and season period, respectively. The same trend label in D refers to SD relation, and so on.

Table 5 quantitatively shows coverages of SG-relations with the non-zero number to industry relations. We can see that the SG-relation can largely cover the industry relations provided by the third-party platform. Among them, SG-relation can cover more than 60% of industry relations in the NYSE market, and even more than 80% in the NASDAQ market. On the other hand, the SGR algorithm adds many stock relations that the third-party platforms fail to provide. In both the NYSE and NASDAQ markets, industry relations contain no more than 5% of SG-relations. Although these stock pairs with SG-relation are not in the same industry, there is a strong correlation between their prices in different periods. These relations can be used as important information for stock price prediction. SG-relations generated by different trading data have different coverage rates for industry relations. Of these, the SG-relation corresponding to the daily price has the highest coverage, and the coverage of other trading data decreases with the growth of the period. Figure 6 shows the rule more intuitively. This phenomenon reveals that the short-term prices of companies in the same industry are correlated, while the long-term price correlation can reflect other relationships.

**Table 5:** SG-relations and industry relations cover each other on NYSE and NASDAQ. There are only SG-relations with a non-zero number. Thus, RD and RW are removed.

	NYSE		NADSAQ	
SG-Relations	Number	Coverage	Number	Coverage
SD	8,075	0.444	2,010	0.622
SW	4,701	0.293	1,148	0.403
SM	2,111	0.16	618	0.214
RM	12	0	3	0.005
SS	1,656	0.09	773	0.179
RS	247	0	56	0.005
Total	13,381	0.624	3,305	0.816
industry relations	721	0.034	201	0.05

### Study on back-testing return of TRMF

**Figure 7:**
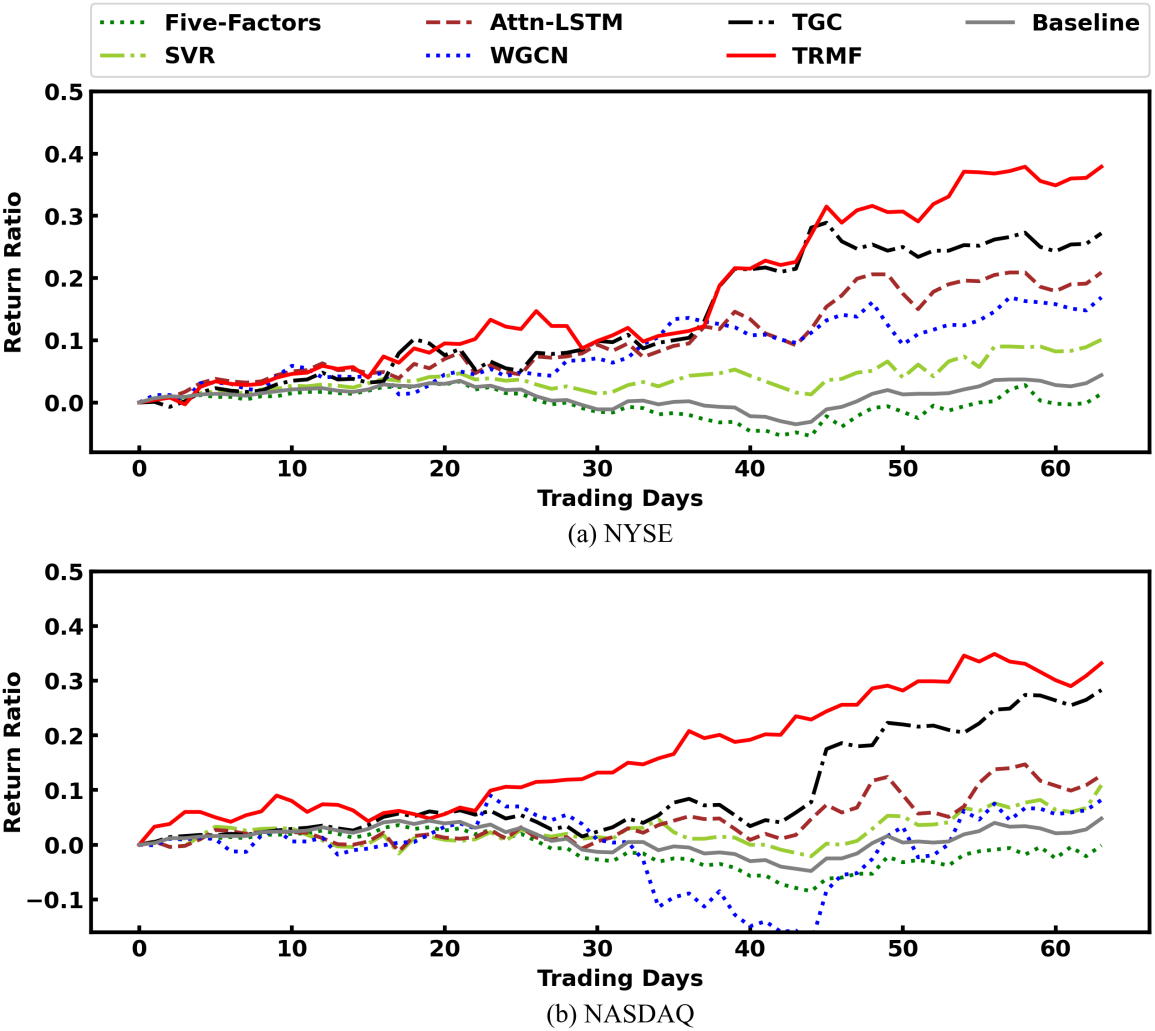
Comparison of cumulative rate of return among models.

Figure 7 presents the cumulative rate of return of all models. Their performance follows the order of TRMF > TGC > Attn-LSTM > WGCN > SVR > Baseline > Five-Factors. And most of the time, TRMF is on top. Therefore, TRMF can bring maximum benefits to investors in the experimental environment.

Table 6 gives the results of each experimental model under four evaluation metrics. We can see that TRMF model outperforms comparison models on most metrics. First, we found both TRMF and TGC outperforms traditional Five-Factors model, SVR, and Attn-LSTM on two markets with great performance *w*.*r*.*t*. IRR, MDD, and SR. It varifies that incorporating stock relation information into the model can significantly improve the stock price prediction. In terms of MDD, the prediction of both TRMF and TGC are significantly smaller than those of other comparable models and benchmark indices. Both them integrate stock relations into the prediction. Therefore, we hypothesized that the relation category factor could effectively reduce the drawdown because the pricing of related stocks gives investors confidence and reduces the overselling situation because of short-term market sentiment. Furthermore, TRMF’s performance was better than the TGC using industry relations, which may be related to the higher number of SG-relations. This indicates that our proposed SG-relations can capture more relation information to enhance the robustness of model predictions. However, TRMF failed to reach the best performance *w*.*r*.*t*. MSE, though it was still the second of all models. The reason could be because TRMF has the most parameters. The WGCN, which only uses relation information, is inferior to the Attn-LSTM which only uses time information in terms of IRR and SR, and it cannot achieve high prediction accuracy with respect to MSE. Therefore, the time series information of stocks is more important, and the stock prices of different stocks retain a large degree of autonomy.

**Table 6:** Performance comparison among models.

	NYSE				NADSAQ			
Model	MSE	IRR	MDD	SR	MSE	IRR	MDD	SR
Five-Factors	7.33e−4	0.014	8.2%	0.011	8.27e−4	0.001	10.3%	0.001
SVR	5.21e−4	0.101	5.9%	0.255	6.32e−4	0.109	7.2%	0.166
Attn-LSTM	2.99e−4	0.214	5.2%	0.237	4.63e−4	0.131	6.5%	0.326
WGCN	3.15e−4	0.172	5.9%	0.172	4.87e−4	0.117	24.9%	0.153
TGC	3.13e−4	0.271	4.6%	0.345	4.37e−4	0.282	4.7%	0.335
TRMF	3.12e−4	0.385	3.2%	0.544	4.41e−4	0.334	4.4%	0.492
Baseline	7.33e−4	0.014	8.2%	0.011	8.27e−4	0.001	10.3%	0.001

## Conclusions

A stock’s future price is affected by its historical trading data and the related stocks. Therefore, in this article, we expected to combine stock relations with multiple information for the price prediction. To solve the problem, we proposed a TRMF model. The core of the TRMF model is an algorithm for richly generating stock relations, named SGR algorithm, which uses trading data to mine multiple SG-relations automatically. Experiments based on data from the NYSE and NASDAQ demonstrated the effectiveness of our solution. The results show that SG-relations can cover more than 60% of industry relations from the third-party platform. TRMF outperformed SVR, Attn-LSTM, and TGC with higher IRR, SR and lower MDD.

We proved that the correlations of price fluctuations in different periods reflect stock relations. But other transaction dimensions, such as trading volume, turnover, and others, are not discussed in the paper. Therefore, in the future, we will investigate the potential of extending the self-generating relation algorithm to other stock trading data to gain more effective relationship information and improve stock price predictions.

## Supplemental Information

10.7717/peerj-cs.1057/supp-1 Supplemental Information 1 Source code of TRMF model with exprimental dataset Folders Data, Model and ZBacktest represent experimental data, model and backtesting experimental process respectively. Hyperparameters are collected in the A_Global file.

## Appendix A. Experimental Stocks

### NYSE

A.N, AAP.N, ABBV.N, ABC.N, ABT.N, ACN.N, ADM.N, AEE.N, AFL.N, AIG.N, AIZ.N, AJG.N, ALB.N, ALK.N, ALL.N, ALLE.N, AME.N, AMP.N, AMT.N, ANET.N, ANTM.N, AON.N, AOS.N, APD.N, APH.N, APTV.N, ARE.N, ATO.N, AVB.N, AVY.N, AWK.N, AXP.N, AZO.N, BA.N, BAC.N, BAX.N, BBWI.N, BBY.N, BDX.N, BEN.N, BF_B.N, BIO.N, BK.N, BLK.N, BLL.N, BMY.N, BR.N, BRK_B.N, BRO.N, BSX.N, BWA.N, BXP.N, C.N, CAG.N, CAH.N, CAT.N, CB.N, CBRE.N, CCI.N, CCL.N, CE.N, CF.N, CFG.N, CHD.N, CI.N, CL.N, CLX.N, CMA.N, CMG.N, CMI.N, CMS.N, CNC.N, CNP.N, COF.N, COO.N, COP.N, CPB.N, CRL.N, CRM.N, CTLT.N, CTRA.N, CVS.N, CVX.N, D.N, DAL.N, DE.N, DFS.N, DG.N, DGX.N, DHI.N, DHR.N, DIS.N, DLR.N, DOV.N, DPZ.N, DRE.N, DRI.N, DTE.N, DUK.N, DVA.N, DVN.N, ECL.N, ED.N, EFX.N, EIX.N, EL.N, EMN.N, EMR.N, EOG.N, EQR.N, ES.N, ESS.N, ETN.N, ETR.N, EW.N, EXR.N, FBHS.N, FDX.N, FE.N, FIS.N, FLT.N, FMC.N, FRC.N, FRT.N, FTV.N, GD.N, GIS.N, GL.N, GLW.N, GM.N, GNRC.N, GPC.N, GPN.N, GPS.N, GS.N, GWW.N, HAL.N, HCA.N, HD.N, HES.N, HIG.N, HII.N, HLT.N, HRL.N, HSY.N, HUM.N, HWM.N, IBM.N, ICE.N, IEX.N, IFF.N, INFO.N, IP.N, IPG.N, IQV.N, IRM.N, IT.N, ITW.N, IVZ.N, J.N, JCI.N, JNJ.N, JNPR.N, JPM.N, K.N, KEYS.N, KMB.N, KMX.N, KO.N, KR.N, KSU.N, L.N, LDOS.N, LEG.N, LEN.N, LH.N, LHX.N, LLY.N, LMT.N, LNC.N, LOW.N, LUV.N, LVS.N, LW.N, LYB.N, LYV.N, MA.N, MAA.N, MAS.N, MCD.N, MCK.N, MCO.N, MDT.N, MET.N, MGM.N, MHK.N, MKC.N, MLM.N, MMC.N, MMM.N, MO.N, MOS.N, MPC.N, MRK.N, MS.N, MSCI.N, MSI.N, MTB.N, MTD.N, NCLH.N, NEE.N, NEM.N, NI.N, NKE.N, NLSN.N, NOC.N, NOW.N, NSC.N, NUE.N, NVR.N, O.N, OKE.N, OMC.N, ORCL.N, OXY.N, PAYC.N, PEAK.N, PEG.N, PFE.N, PG.N, PGR.N, PH.N, PHM.N, PKG.N, PKI.N, PLD.N, PM.N, PNC.N, PNR.N, PNW.N, PPG.N, PPL.N, PRU.N, PSA.N, PSX.N, PVH.N, PWR.N, PXD.N, RCL.N, RE.N, RHI.N, RJF.N, RL.N, RMD.N, ROK.N, ROL.N, ROP.N, RSG.N, RTX.N, SCHW.N, SEE.N, SHW.N, SJM.N, SLB.N, SNA.N, SO.N, SPG.N, SPGI.N, SRE.N, STE.N, STT.N, STZ.N, SWK.N, SYF.N, SYK.N, SYY.N, T.N, TAP.N, TDG.N, TDY.N, TEL.N, TFC.N, TFX.N, TGT.N, TJX.N, TMO.N, TPR.N, TRV.N, TSN.N, TT.N, TXT.N, TYL.N, UDR.N, UHS.N, UNH.N, UNP.N, UPS.N, URI.N, USB.N, V.N, VFC.N, VLO.N, VMC.N, VNO.N, VTR.N, VZ.N, WAB.N, WAT.N, WEC.N, WELL.N, WFC.N, WHR.N, WM.N, WMB.N, WMT.N, WRB.N, WRK.N, WST.N, WU.N, WY.N, XOM.N, XYL.N, YUM.N, ZBH.N, ZTS.N

### NASDAQ

AAL.O, AAPL.O, ABMD.O, ADBE.O, ADI.O, ADP.O, ADSK.O, AEP.O, AKAM.O, ALGN.O, AMAT.O, AMGN.O, AMZN.O, ANSS.O, APA.O, ATVI.O, AVGO.O, BIIB.O, BKNG.O, CDNS.O, CDW.O, CERN.O, CHRW.O, CHTR.O, CINF.O, CMCSA.O, CME.O, COST.O, CPRT.O, CSCO.O, CSX.O, CTAS.O, CTSH.O, CTXS.O, CZR.O, DISCA.O, DISCK.O, DISH.O, DLTR.O, DXCM.O, EA.O, EBAY.O, EQIX.O, EXC.O, EXPD.O, EXPE.O, FANG.O, FAST.O, FB.O, FFIV.O, FISV.O, FITB.O, FTNT.O, GILD.O, GOOG.O, GOOGL.O, HAS.O, HOLX.O, HON.O, HSIC.O, HST.O, IDXX.O, ILMN.O, INCY.O, INTC.O, INTU.O, IPGP.O, ISRG.O, JBHT.O, JKHY.O, KHC.O, KLAC.O, LKQ.O, LNT.O, LRCX.O, MAR.O, MCHP.O, MDLZ.O, MKTX.O, MNST.O, MPWR.O, MSFT.O, MU.O, NDAQ.O, NFLX.O, NLOK.O, NTAP.O, NTRS.O, NVDA.O, NXPI.O, ODFL.O, ORLY.O, PAYX.O, PCAR.O, PEP.O, PFG.O, POOL.O, PTC.O, PYPL.O, QCOM.O, QRVO.O, REG.O, REGN.O, ROST.O, SBAC.O, SBUX.O, SIVB.O, SNPS.O, STX.O, SWKS.O, TECH.O, TER.O, TMUS.O, TRMB.O, TROW.O, TSCO.O, TSLA.O, TTWO.O, TXN.O, UAL.O, ULTA.O, VIAC.O, VRSK.O, VRSN.O, VRTX.O, WBA.O, WDC.O, WLTW.O, WYNN.O, XEL.O, XLNX.O, XRAY.O, ZBRA.O, ZION.O

## Additional Information and Declarations

### Data Availability

The following information was supplied regarding data availability: Raw data and code are available in the Supplemental Files.
